# Gaze Following and Attention to Objects in Infants at Familial Risk for ASD

**DOI:** 10.3389/fpsyg.2019.01799

**Published:** 2019-08-20

**Authors:** Janet P. Parsons, Rachael Bedford, Emily J. H. Jones, Tony Charman, Mark H. Johnson, Teodora Gliga

**Affiliations:** ^1^Centre for Brain and Cognitive Development, Birkbeck, University of London, London, United Kingdom; ^2^Biostatistics Department, Institute of Psychiatry, Psychology & Neuroscience, King’s College London, London, United Kingdom; ^3^Department of Psychology, Institute of Psychiatry, Psychology & Neuroscience, King’s College London, London, United Kingdom; ^4^Department of Psychology, University of Cambridge, Cambridge, United Kingdom; ^5^Department of Psychology, University of East Anglia, Norwich, United Kingdom

**Keywords:** gaze following, infants, familial risk, ASD, eye-tracking

## Abstract

Reduced gaze following has been associated previously with lower language scores in children with autism spectrum disorder (ASD). Here, we use eye-tracking in a controlled experimental setting to investigate whether gaze following and attention distribution during a word learning task associate with later developmental and clinical outcomes in a population of infants at familial risk for ASD. Fifteen-month-old infants (*n* = 124; *n* = 101 with familial risk) watched an actress repeatedly gaze toward and label one of two objects present in front of her. We show that infants who later developed ASD followed gaze as frequently as typically developing peers but spent less time engaged with either object. Moreover, more time spent on faces and less on objects was associated with lower concurrent or later verbal abilities, but not with later symptom severity. No outcome group showed evidence for word learning. Thus, atypical distribution of attention rather than poor gaze following is a limiting factor for language development in infants at familial risk for ASD.

## Introduction

Typically developing infants are sensitive to others’ gaze from birth (Batki et al., 2000; Farroni et al., 2002). Over the first year they follow gaze first reflexively (Hood et al., 1998; Farroni et al., 2000, 2004) and then learn its referential function (Woodward, 2003; Csibra and Volein, 2008; Senju et al., 2008). Being able to follow someone’s gaze, and jointly attend to objects, is thought to provide a key mechanism by which infants acquire a vocabulary (Baldwin, 1991, 1993; Schafer and Plunkett, 1998; Houston-Price et al., 2006) and many studies have associated joint attention ability with later vocabulary growth (Carpenter et al., 1998; Morales et al., 1998; Charman, 2003; Brooks and Meltzoff, 2008).

Children with autism spectrum disorder (ASD) often have poor joint attention, evidenced by reduced gaze following in naturalistic situations (e.g., Dawson et al., 2004), and this has been highlighted as one of the most reliable and consistent indicators of ASD during childhood (e.g., Baron-Cohen et al., 1996; Charman, 2003). Given that the rate of learning difficulty is often high in children with ASD (∼55%; Charman et al., 2011) and there is frequent language delay (e.g., Charman et al., 2003), studies have suggested that poor language in ASD may be explained, in part, by difficulties with engaging in joint attention (e.g., Mundy et al., 1994; Pickard and Ingersoll, 2015). For example, in their study of children between 22 to 93 months of age, Pickard and Ingersoll used the Early Social Communication Scales, a play-based structured assessment that captures both the child’s initiating and responding to joint attention, to show that failing to follow someone’s gaze or pointing to an object were best predictors of concurrent language. In addition, an intervention targeting joint attention in children with ASD yielded better expressive language outcomes when compared to an intervention increasing symbolic play (Gulsrud et al., 2014).

There are several reasons why children with ASD may struggle to use joint attention for learning language. Firstly, they may not correctly or consistently follow someone’s gaze to the object they are labeling. This could be because they do not spend enough time looking at faces to notice or process the gaze shifts. Alternatively, despite looking at faces and eyes, they still may not shift their gaze in the same direction as the person communicating with them. It could also be that, despite correctly following gaze, they do not spend enough time on the gazed at object to learn about it. Looking less toward the gazed at object may also reflect poor understanding of the referential nature of gaze. That is, word learning could fail not because there was insufficient time dedicated to encoding object properties, but because unlike typically-developing children (Gliga and Csibra, 2009), children with ASD may have a reduced appreciation of the referential link between the uttered word and the gazed at object.

Recently, eye-tracking studies have allowed a detailed quantification of attention distribution during joint attention episodes, thus making it possible to reveal the different sources of atypicality mentioned above. Eye-tracking studies investigating how young children with ASD respond to gaze cues, are summarized in Tables 1.1–1.3. We review studies of children up to 4 years of age, because beyond this age, children with a diagnosis of ASD are likely to take part in intervention programs which may affect performance in experimental studies. Since it is important to investigate the ability to respond to referential cues when it most contributes to vocabulary growth (Morales et al., 2000) we give special attention to longitudinal studies of infants at familial risk for ASD, which study infants during their first 2 years of life. This population has a higher likelihood of developing ASD themselves (∼20%, Ozonoff et al., 2015; general population ∼1–2%). A further 20% will exhibit subthreshold symptoms of ASD or developmental delay (Messinger et al., 2013).

**TABLE 1.1:** Results for attention to the face from eye-tracking studies exploring joint attention in young children with ASD or at-risk for ASD.

**Paying attention to faces**				
**Article**	**Participants**	**Measure**	**ASD vs. others^1^**	**Additional information^2^**
Chawarska et al., 2013	High-risk^∗∗^, LR 6 mo.	F/Scene	Less	Conditions include dyadic bids and gaze shifts
		E/Scene	Same	

Jones and Klin, 2013	High-risk^∗∗^, LR			During dyadic bids
	2 mo. to 6 mo.	E/Scene	More	
	6 mo. to 24 mo.	E/Scene	Less	

Nyström et al., 2017	High-risk^∗^, LR			LIVE interaction (not screen-based)
	10 mo.	F/Scene	Less	200–700 ms after mutual gaze
		F/Scene	Same	Across the whole session

Thorup et al., 2016, 2018	High-risk^∗^, LR			LIVE interaction (not screen-based)
	10 mo.	F/Scene	Same	No difference in the time to engage the actor

This study	High-risk^∗∗^, LR 15 mo.	F/Scene	Same	During gaze shifts

Chawarska et al., 2012	ASD, TD 13–25 mo.	F/Scene	Less	During dyadic bids
		F/Scene	Same	During gaze shifts

Billeci et al., 2016	ASD, TD 18–30 mo.	F/Scene	Same	During gaze shifts
		F/Scene	More	When toddler initiates joint attention

Jones et al., 2008	ASD, TD 24–27 mo.	E/Scene	Less	During dyadic bids

Vivanti et al., 2017	ASD, TD 48 mo.	F (not scaled)	Less	During gaze shifts

*Studies were chosen where attention had to be distributed between faces and other objects in the scene/background. Studies are organized by participant age (youngest to oldest) to highlight any developmental progression. E, eye area; F, whole face area. ^∗^High familial risk studies without diagnostic outcome comparing LR with HR; ^∗∗^High risk studies with analysis by outcome. TD, typically developing; ASD, diagnosed. ^1^‘Less’ indicates ASD or high familial risk participants had lower values than typically developing/low familial risk participants, ‘more’ indicates ASD or high familial risk participants had greater values. ^2^This column contains additional relevant information regarding experimental conditions.*

**TABLE 1.2:** Direction of first look results from eye-tracking studies exploring joint attention using gaze following in young children with ASD or at-risk for ASD.

**Direction of first look in response to gaze shifts**				
**Article**	**Participants**	**Measure**	**ASD vs. others^1^**	**Additional information^2^**
Bedford et al., 2012	High-risk^∗∗^, LR 7 and 13 mo.	R/(R + D + O + F)	Same	

Thorup et al., 2016	High-risk^∗^, LR 10 mo.			LIVE interaction (not screen-based)
		R-D	Same	Response to Eye + Head better than Eyes only for HR but not LR group
		R-D/(R + D)	Same	Both groups above chance

Nyström et al., 2019	High-risk^∗∗^, LR 10 mo.	R-D	Same	LIVE interaction (not screen-based)

This study	High-risk^∗∗^, LR 15 mo.	(R-D)/(R + D)	Same	All above chance
		R/(R + D + O + F)	Same	

Billeci et al., 2016	ASD, TD 18–30 mo.	(R–D)/(R + D)	Same	Chance comparison not reported

Gliga et al., 2012	High-risk^∗∗^, LR 36 mo.	R/(R + D)	Same	All above chance

Falck-Ytter et al., 2015	ASD, TD 41 mo.	R-D	Same	

Vivanti et al., 2017	ASD, TD 48 mo.	R/(R + D)	Less	Chance comparison not reported
			Same	After excluding trials with face dwell time during gaze shift < 100 ms

Gillespie-Lynch et al., 2013	ASD, TD 28–79 mo.	R-D	Less	

Thorup et al., 2017	ASD, TD 38–115 mo.	R/(R + D)	Same	All above chance

*Studies are organized by participant age (youngest to oldest) to highlight any developmental progression. R, object referenced; D, distractor object; F, whole face area; O, other areas of screen. ^∗^High familial risk studies without diagnostic outcome comparing LR with HR; ^∗∗^High familial risk studies with analysis by outcome. TD, typically developing; ASD, diagnosed. ^1^‘Less’ indicates ASD or high familial risk participants had lower values than typically developing/low familial risk participants. ^2^This column contains additional relevant information regarding experimental conditions or comparisons to chance (where appropriate).*

**TABLE 1.3:** Results for attention engagement with objects from eye-tracking studies exploring joint attention using gaze following in young children with ASD or at-risk for ASD.

**Engaging attention with gazed at objects**				
**Article**	**Participants**	**Measure**	**ASD vs. others^1^**	**Additional information^2^**
**Engaging attention with gazed at objects**				
Bedford et al., 2012	High-risk^∗∗^, LR	R/(R + D + O + F)		
	7 mo.		Same	
	13 mo.		Less	

This study	High-risk^∗∗^, LR 15 mo.	(R-D)/(R + D)	Same	All groups above chance
		R/(R + D + O + F)	Less	

Billeci et al., 2016	ASD, TD 18–30 mo.	R/(R + D + O + F)	Same	

Gliga et al., 2012	High-risk^∗∗^, LR 36 mo.	R/(R + D)	Same	All groups above chance

Falck-Ytter et al., 2015	ASD 41 mo. TD 21 mo.	R-D First fixation	Less	

Vivanti et al., 2017	ASD, TD 48 mo.	R (not scaled)	Less	
		R (not scaled)	Trending less	After excluding trials with dwell time on face < 100 ms during gaze shift

Thorup et al., 2017	ASD, TD 38–115 mo.	R/(R + D) First fixation	Less	When referent was not an object of high interest, i.e., a pot plant
		R/(R + D) First fixation	Same	When referent was an object of high interest, i.e., trains/vehicles

*Studies are organized by participant age (youngest to oldest) to highlight any developmental progression. R, object referenced; D, distractor object; F, whole face area; O, other areas of screen. ^∗^High familial risk studies without diagnostic outcome comparing LR with HR; ^∗∗^High familial risk studies with analysis by outcome. TD, typically developing; ASD, diagnosed. ^1^‘Less’ indicates ASD or high familial risk participants had lower values than typically developing/low familial risk participants. ^2^This column contains additional relevant information regarding experimental conditions or comparisons to chance (where appropriate).*

We asked first whether studies found decreased engagement with faces, when children with ASD were presented with scenes in which attention had to be distributed between people and objects. These studies have yielded a mixed picture, with some finding less looking to faces in ASD (Chawarska et al., 2012, 2013; Jones and Klin, 2013), others more looking (Billeci et al., 2016) and yet others no difference between groups (Thorup et al., 2016, 2018). As Table 1.1 suggests, these inconsistencies do not seem to reflect differences in the age of the participants. Some authors have suggested differences between studies may result from variation in the communicative content of the scene, with reduced looking in ASD particularly when the face addresses the child (Shic et al., 2014) or when it establishes mutual gaze (Nyström et al., 2017). One study has directly addressed the question of whether directed communication is particularly problematic (Vernetti et al., 2018). In this study, toddlers could choose between animating (by looking at them) either a video of a person that established eye contact and directly addressed them, or a video of a spinning mechanical toy. There was no difference between those with a later diagnosis of ASD and those without, with all groups choosing to animate and engage longer with the face rather than the toy. Those studies which have analyzed dwell time to the face during gaze following have also failed to find group differences (Chawarska et al., 2012; Billeci et al., 2016; Vivanti et al., 2017), suggesting that poor gaze following in ASD may not be due to insufficient engagement with faces.

During infancy and early toddlerhood, eye-tracking studies are consistent in suggesting that the ability to shift one’s gaze to follow someone else’s gaze direction to an object (henceforth referent) rather than an equally salient distractor, is intact in toddlers with ASD or infants with later ASD, with differences appearing to emerge later in development (see Table 1.2). There is, however, a more mixed picture when studies analyzed the dwell time on objects, with most studies finding decreased looking toward the gazed at objects, but a few finding no differences (see Table 1.3). Some of the inconsistency in findings may reflect differences in the way engagement with objects was measured. Researchers either directly compared time spent on referent versus distractor or contrasted time spent on the referent to time spent on all areas of interest (AOI), including the face or the background. While the former measure directly assesses an understanding of which object is the referent of the gaze, the latter measure also captures infants’ engagement with irrelevant aspects of the scene or differences in looking toward the face. However, no consistent associations between a certain way of measuring engagement with objects and later ASD emerges in this brief review. The only previous study of infants at risk that looked at engagement with objects, found that infants who later developed ASD engaged less with the referent as compared to the whole scene but did not directly compare attention distribution between referent and distractor (Bedford et al., 2012).

Given that gaze following has been suggested as one of the sources of atypical language development in ASD, surprisingly few studies have measured gaze following in the context of word learning. To address existing gaps in the literature and clarify the above inconsistencies in findings, the current study investigated visual behavior during a word learning task in a population of 15-month-olds with older siblings with ASD. We specifically asked whether atypicalities previously reported for infants later diagnosed with ASD reflect poor following or understanding of gaze direction, in which case we would find differences in measures directly comparing attention to the referent and the distractor; alternatively, they may reflect differences in attention distribution across the whole scene which may emerge when dwell time to the face or other parts of the screen are investigated. To clearly distinguish these two sets of measures, we refer to the former as gaze following and the latter as attention distribution. In addition to comparing performance between the four outcome groups: low-risk controls (LR), high risk with typical development (HR-TYP), high risk with atypical development (HR-ATYP) and high risk with ASD (HR-ASD), we also investigated the association between experimental variables and continuous measures of ASD traits, language and developmental level. This approach aligns with the recent shift away from the reliance on categorical diagnostic boundaries for research and a move toward the use of continuous measures characterizing individual domains of interest (Insel et al., 2010).

In summary, we predicted that:

(1) HR-ASD infants will show typical gaze following as measured by first look direction, evident as a significant difference between first looks to referent and distractor;
(2) HR-ASD infants will spend significantly less dwell time on the referent than the other groups;
(3) As a consequence of less dwell time spent on the referent, HR-ASD infants would show significantly poorer object-label mapping.

We were unable to make a clear prediction for dwell time on the face since previous studies have been equivocal, reporting both significantly less time and no differences.

## Materials and Methods

### Participants

A cohort of 116 high-risk (HR) (64 males: 52 females) and 27 low-risk (LR) children (14 males: 13 females) participated in the BASIS longitudinal study. All HR children had at least one older sibling with a community clinical diagnosis of ASD. LR controls were full term infants (gestational ages 38–42 weeks), recruited from a volunteer database at the Birkbeck Centre for Brain and Cognitive Development. Families attended four visits at 8, 15, 24, and 36 months. The task analyzed here was run at the 15-month visit (visit 2). Three HR children absent from the 36-month visit were excluded from the analysis. However, two HR children and two LR children absent from the 36-month visit were included in the analysis since outcome could be assessed (see section “Clinical Measures”). An additional 12 HR and 4 LR were excluded based on eye-tracking data availability/quality (see section “Apparatus and Data Preparation” for exclusion procedure). Hence 101 HR and 23 LR infants contributed data to this manuscript. Details regarding the diagnostic screening of the older siblings of these participants are included in the Supplementary Material (S1).

### Clinical Measures

A battery of clinical research measures was administered to all children attending at 36 months; due to non-attendance these measures were unavailable for 7 infants (2 LR and 5 HR). The Autism Diagnostic Observation Schedule – Second Edition (ADOS-2; Lord et al., 2012), a standardized observational assessment, was used to assess current symptoms of ASD. Calibrated Severity Scores for Social Affect and Restricted and Repetitive Behaviors (RRB) were computed (Gotham et al., 2009), which provide standardized autism severity measures that account for differences in module administered, age and verbal ability. The Autism Diagnostic Interview – Revised (ADI-R; Le Couteur et al., 2003), a structured parent interview, was completed with parents/caregivers. Standard Algorithm scores were completed for Reciprocal Social Interaction (Social), Communication and Restricted, Repetitive and Stereotyped Behaviors and Interests (RRB). These assessments were conducted without blindness to risk-group status, by or under the close supervision of clinical researchers (i.e., psychologists, speech, and language therapists) with demonstrated research-level reliability. We used the Early Learning Composite score of the Mullen Scales of Early Learning (MSEL; Mullen, 1995) to obtain a standardized measure of developmental level at every visit.

Experienced researchers (TC, GP, CC) reviewed information on ASD symptomology (ADOS-2, ADI-R), adaptive functioning (*Vineland Adaptive Behavior Scale-II*, Sparrow et al., 2015) and development (MSEL; Mullen, 1995) for each HR and LR child to ascertain ASD diagnostic outcome according to DSM-5 (American Psychiatric Association, 2013). Of the 101 HR participants contributing data for this study, 12 (10 boys, 2 girls) met criteria for ASD (HR-ASD). A further 26 participants (18 boys, 8 girls) did not meet ASD criteria but were not considered typically-developing, due either to (a) scoring above ADI-R cut-off for ASD (Risi et al., 2006) and/or scoring above ADOS-2 cut-off for ASD (*n* = 12), or (b) scoring less than 1.5 SD below the population mean on the Mullen Early Learning Composite (<77.5) or on the Mullen Expressive Language or Receptive Language subscales (<35) (*n* = 9), or meeting both of the points (a) and (b) above (*n* = 5). These participants therefore comprised a HR sub-group, who did not meet clinical criteria for ASD but presented with other atypicalities (HR-ATYP). The remaining 63 HR participants (27 boys, 36 girls) were typically developing (HR-TYP). None of the 23 LR children contributing data for this study (13 boys, 10 girls) met DSM-5 criteria for ASD and none had a community clinical ASD diagnosis.

Note, for four of the seven children absent at the 36-month visit, 2 LR and 1 HR were classified as typically-developing on the basis of typical development at the previous three visits and 1 HR infant was classified as HR-ASD both on the basis of behavior at previous visits and by confirmation through local diagnosis.

### Stimuli and Procedure

Participants saw teaching and test trials which used two object pairs (four distinct objects). Four pseudo-words were used to label the objects (kobe, toma, sefo, dax) and mappings between a particular object and word were fixed (object pair 1: kobe/toma; object pair 2: sefo/dax). For each word, infants were presented with two *teaching trials*, which only differed in the left/right position of the objects. Each *teaching trial* (approximately 11 s) began with direct gaze from an actress and a greeting (‘hello’), the actress exclaimed ‘look,’ shifted gaze toward one object (the referent), labeled it (e.g., ‘a kobe’) and turned back to direct gaze (see Supplementary Video 1). Two further gaze shifts labeling the same object were completed with differing exclamations during direct gaze then labeling whilst the actress looked at the referent (‘wow, a kobe,’ ‘see, a kobe’). The trial ended with the actress looking at the referent after the third gaze shift. Each *testing trial* (approximately 8 s) showed the referent and its paired object as a distractor, without the actress present. For one of the object pairs, each object was labeled then immediately followed by a test trial (one-word test trials); for the other object pair both objects were labeled before being followed by the corresponding test trials (two-word test trials). Two-word test trials were more difficult since the infant could only succeed if they associated the words and the objects. When only one object in the pair was labeled, infants may perform correctly during testing (i.e., look longer at the referent of the label) by simply remembering which object had been labeled before, thus without needing to remember the association between that object and the label. The word used in teaching to refer to the gazed at object, was heard four times in the one-word test trials and three times in the two-word test trials. The first presentation of the word was 2.5 s after test trial onset in one-word test trials and 2.75 s in two-word test trials. These differences were the result of experimental error and not deliberate.

**FIGURE 1:**
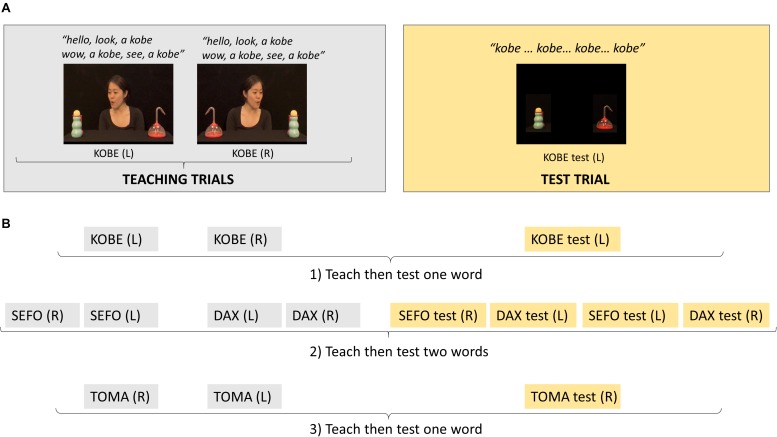
**(A)** Example screen shots from the teaching and test trials for one of the words learnt; for each word, the first teaching trial had the referent object positioned on one side of the screen, in this example, left side [KOBE (L)] and on the opposite side in the second teaching trial [KOBE (R)]. **(B)** The order in which teaching and test trials for different words were presented which created one-word tests (1:KOBE and 3:TOMA) and two-word tests (2:SEFO, DAX); R, referent on right side of screen; L, referent on left side of screen.

Figure 1 illustrates the sequence of teaching and test trials. Infants saw these in a fixed order. The first two teaching trials labeled one of the objects from the first pair, one trial with object positioned on the left of the screen then one with it on the right, followed by one test trial. The next four teaching trials labeled both objects in the second pair, once for each object in each position, followed by four tests trials, one for each object in each position. Finally, the last two teaching trials labeled the second object in the first pair, followed by one test trial. This meant that objects presented as referents in the first four trials became distractors in the following four trials. This order was motivated by the need to temporally separate the teaching/test trials for the objects in pair 1 so that they both acted as one-word tests.

Infants were seated on their parents’ lap at approximately 60 cm from a Tobii T120 eye tracker screen (Tobii Technology, Stockholm, Sweden). A five-point calibration routine was run. The experiment began when at least four points were marked as calibrated for each eye. The infant’s behavior was monitored by a video camera placed above the eye-tracker monitor. Stimuli were presented with Tobii Studio software. Between teaching and test trials and also between the two-word test trials, the child’s attention was re-directed to the center of the screen using two central bright-colored shapes displayed consecutively, each for 500 ms.

### Apparatus and Data Preparation

Data was recorded at 60 Hz using the Tobii T120 eye tracker (Tobii Technology, Stockholm, Sweden). It was extracted from Tobii Studio into raw data files using the ClearView filter which identified fixations as stable gaze within a 100-pixel radius, for at least 60 ms duration. This distinguished fixations from saccades and other random noise such as imperfections in system set-up, tremor, and micro-saccades in eye movements (Olsen, 2012).

Areas of interest were defined separately around the face, referent and distractor for teaching trials and around the referent and distractor for test trials. Fixation points (X,Y coordinates) were assigned to AOIs using Matlab R2016b (MathWorks, Inc., Natick, MA, United States). Where samples were missing for less than 200 ms and samples before and after indicated the same AOI, they were set to that AOI. This threshold was used since it is unlikely the infant could have shifted their gaze away and back during that time given the minimum time taken to program a saccade is 100–130 ms (Inhoff and Radach, 1998; Radach et al., 1999). Finally, data was summarized per participant in MatLab then transferred for analysis in SPSS (version 23, IBM Corp, 2015).

### Data Reduction

From the cohort of 143 infants (116 HR and 27 LR) taking part in the BASIS longitudinal study, 3 HR children were excluded as they had no outcome recorded. Outcomes for the remaining 113 HR children were: HR-TYP *n* = 64, HR-ATYP *n* = 32, HR-ASD *n* = 17. However, 16 infants (12 HR and 4 LR) did not contribute eye-tracking data for this study, one because they did not attend the lab visit (HR-ATYP), three were excluded due to eye-tracking equipment failure (HR-ATYP *n* = 1, HR-ASD *n* = 2) and for 12 others the task was interrupted because of fussiness (LR *n* = 4, HR-TYP *n* = 1, HR-ATYP *n* = 4, HR-ASD *n* = 3). Hence data from 101 HR (HR-TYP *n* = 63, HR-ATYP *n* = 26, HR-ASD *n* = 12) and 23 LR infants was analyzed. Descriptive characteristics and clinical measures by group for these infants are presented in Table 2.

**TABLE 2:** Detailed characterization for participants that contributed data with standard deviations.

	**High risk**			**Low risk (LR)**	***p*-values**
	**HR-ASD**	**HR-ATYP**	**HR-TYP**		
*N*	12	26	63	23	–
M:F	10:2	18:8	27:36	13:10	–
**15 months**					
Age in mths (SD)	14.83(1.03)	14.88(1.03)	14.92(0.94)	15.04(0.88)	0.834^b^
MSEL ELC^1^	82.17(10.55)	93.04(14.88)	97.69(11.96)^a^	103.00(15.59)^a^	<0.001
CDI words^1^ understood	43.64(46.00)	75.04(56.68)	107.18(73.10)^a^	102.70(74.89)^a^	0.006^b^
**24 months**					
Age in mths (SD)	26.90(3.14)	25.86(2.06)	26.11(1.83)	24.55(0.89)^a^	0.002^b^
MSEL ELC^2^	79.25(20.28)	94.71(23.32)	104.34(15.41)^a^	115.65(15.22)^a^	<0.001
CDI words^2^ understood	174.20(101.97)	324.52(178.49)	423.48(154.19)^a^	476.30(125.62)^a^	<0.001
**36 months**					
*N*^3^	11	26	62	21	–
Age in mths (SD)	38.91(1.76)	38.77(1.88)	38.87(1.41)	38.81(1.50)	0.904^b^
MSEL ELC	83.73(25.44)	87.04(25.89)	114.19(15.73)^a^	119.57(15.46)^a^	<0.001
ADI-social	12.00(5.00)	2.54(2.55)^a^	1.48(2.01)^a^	1.05(1.60)^a^	< 0.001^b^
ADI-communication	11.73(4.41)	3.81(3.92)^a^	1.73(2.20)^a^	0.48(1.12)^a^	< 0.001^b^
ADI-RRB	5.36(2.73)	0.92(1.29)^a^	0.47(0.92)^a^	0.10(0.30)^a^	< 0.001^b^
ADOS-social affect	4.18(3.25)	4.58(2.50)	1.60(0.76)^a^	2.76(2.05)	< 0.001^b^
ADOS-RRB	6.36(1.63)	4.85(2.68)	3.34(2.34)^a^	3.24(2.23)^a^	0.001^b^
SRS total *t*-score	68.18(12.67)	48.52(10.11)	45.34(8.66)^a^	41.90(4.22)^a^	< 0.001^b^

*MSEL ELC Mullen Scales of Early Learning Early Learning Composite; CDI words understood (MacArthur-Bates Communicative Development Inventory) was derived by summing words understood only and words understood and spoken; ADI (The Autism Diagnostic Interview – Revised), a structured parent interview; ADOS (The Autism Diagnostic Observation Schedule), a standardized observational assessment, values are calibrated severity scores; SRS (Social Responsiveness Scale), a parent questionnaire, the value reported is the total t-score. ^*a*^Indicates significant differences with the HR-ASD group. ^*b*^Indicates measures not normally distributed for which outcome group comparisons used the non-parametric Kruskal–Wallis H test. ^1^15 month Mullen is missing for 1 participant (HR-TYP); 15 month CDI is missing for 4 participants (1 HR-TYP, 2, HR-ATYP, 1 HR-ASD). ^2^24 month Mullen is missing for 7 participants (2 HR-TYP, 4 HR-ATYP); 24 month CDI is missing for 17 participants (3 LR, 7 HR-TYP, 5 HR-ATYP, 2 HR-ASD). ^3^Four children whose data is included in analysis did not attend at 36 months: 2 LR; 1 HR sibling classified HR-TYP, 1 HR sibling classified HR-ASD (see section “Clinical Measures”).*

We analyzed two looking behaviors: the direction of infants’ *first looks* after the actress’ first gaze shift during the teaching trials and infants’ *dwell times* on regions of interest, during both the teaching and the test trials.

#### First Looks

This was defined by the direction of the infant’s first gaze shift in response to seeing the actress’ first gaze shift to one of the two objects, i.e., between 2750 and 5400 ms from the beginning of the teaching trial. Trials were considered valid provided infants’ gaze was on the face within 200 ms from the start of the actress’ first gaze shift. Behavior for valid trials was classified as (1) directing their first look to the referent and (2) to the distractor.

#### Dwell Time

Dwell time was defined as the number of samples in which gaze was within a particular AOI. Two proportional dwell time measures were created: a direct comparison between referent and distractor (R-D)/(R + D); and broader distribution measures for each AOI relative to the total number of samples on the screen. For the teaching trials, proportion of dwell time on the face was calculated for the period from the actress initiating the dyadic bid to the start of the first gaze shift (1000–2750 ms) then AOI dwell time proportions for each AOI were calculated from the beginning of the first gaze shift to the end of the trial. For some analyses (see below), AOI dwell time proportions were calculated separately for each of the actresses’ gaze shifts: shift 1 (2750–5400 ms), shift 2 (5400–8050 ms), shift 3 (8050–11670 ms). Since we wanted to explore general patterns of attention distribution over time during gaze following, we included all teaching trials in our analysis, even when the infant did not start on the face at the beginning of the trial. The more liberal criteria for the dwell time measure (compared to the first look) was used because data was taken across the whole trial which involved the actress making multiple gaze shifts.

### Analytical Approach

Across parametric analyses we covaried data quality (%samples detected) and age in months, and weighted by number of trials. We also tested these variables for outcome group differences in each analysis. Non-parametric Kruskal–Wallis tests indicated no outcome group differences in any analysis for data quality, age, or number of trials contributing.

We began by checking for any outcome group differences in dwell time to the actress’ face just prior to the first gaze shift, during the dyadic interaction. If such differences were present, this might explain outcome differences in first looks and/or attention distribution during gaze shifts, especially for the first gaze shift. The percentage dwell time on the face in this time period was not normally distributed, hence a Kruskal–Wallis H test was used.

For first looks and dwell time during gaze shifts, we first directly compared looking to the referent and the distractor, calculated as the difference between the measure taken for the referent and the distractor, scaled by their sum, i.e., (R-D)/(R + D), as in other studies mentioned in Tables 1.2, 1.3. We will refer to these measures as gaze following. Values range from −1, where first looks or dwell time are directed exclusively to the distractor, to +1 where first looks or dwell time are directed exclusively to the referent. The chance level is zero. Since this measure for first looks was not normally distributed, non-parametric tests were used to make chance and outcome group comparisons (Wilcoxon signed-rank test and Kruskal–Wallis H test respectively). This measure was normally distributed for dwell times, hence parametric tests were used for chance and outcome group comparisons (one sample t-test and analysis of covariance respectively).

We then analyzed the broader distribution of dwell time during gaze shifts; for this analysis dwell time to referent, distractor, the face and the background were scaled by overall screen time (see also Bedford et al., 2012). Since AOI dwell time proportions are correlated, a generalized estimating equation (GEE) approach with an unstructured working correlation matrix was chosen. The analysis used a Gaussian model with identity link (participant id) between predictors and expected proportions and with AOI as a within-participant and outcome group as a between-participant variable. Since changes in performance may occur with the repetition of the actress’ gaze shifts (see Figure 1), time-segment (shift1, shift2, shift3) was also added as a within-participant variable.

We also performed additional analyses which more closely followed the approach taken by Bedford et al. (2012). These are included in the Supplementary Material: the analysis of the broader distribution of first looks to each AOI (S3) and the analysis of dwell time in teaching trials excluding those in which infants did not make a congruent first look (S4).

Between the 8 and 15-month visits, 51 of the high-risk families took part in a randomized controled trial (RCT) of parent-mediated intervention (Green et al., 2015), with an additional five families enrolled in a similar non-RCT intervention (Green et al., 2013). All preliminary analyses included two binary terms as predictors: treatment (non-treated vs. treated) and recruitment (not recruited for the intervention trials vs. recruited for intervention trial, irrespective of treatment status). As we were not interested in investigating the effects of treatment and recruitment, the analyses were completed only to examine whether the inclusion of these factors would alter the significance of results. Recruitment did not change the significance level of any effects reported. Treatment changed the significance of two results and this is reported where relevant (see section “Attention to the Actress’ Face Relative to Screen Time”; and Supplementary Material, S2.2).

Finally, we asked whether experimental dwell times during teaching trials associated with phenotypic measures. First, we asked if dwell time measures associated with continuous measures of ASD symptoms. Three different measures for ASD symptoms were used, each capturing ASD traits in a different manner: parental interview, Autism Diagnostic Interview (ADI; Le Couteur et al., 2003); observational, Autism Diagnostic Observation Schedule (ADOS-2, Lord et al., 2012); and parent report questionnaire, Social Responsiveness Scale (SRS; Constantino and Gruber, 2005). Then we looked at associations with developmental measures, including two language measures, the Communicative Development Inventory (CDI; Fenson et al., 2007), measured concurrently and at 24 months, and combined verbal scales (receptive and expressive language) of the Mullen Scales of Early Learning (MSEL; Mullen, 1995), measured concurrently and at 36 months. When both variables were normally distributed, we used the Pearson correlation coefficient to benefit from greater power; when one or both variables were not normally distributed, we used Kendall’s tau. Kendall’s tau was used in preference to Spearman’s rho because it deals more accurately with tied ranks, frequent in our data, and provides a superior estimate of the correlation in the population, allowing more accurate generalization (Howell, 1997).

## Results

On analyzing data from test trials, we found no evidence of object-label mapping (‘word learning’) in any outcome group. This held for both one-word and two-word tests. A detailed description of this analysis is given in the Supplementary Material (S2). Therefore, our third hypothesis could not be tested. In contrast, outcome group differences were found in data from teaching trials and these are reported in detail in the following sections.

### Looking to the Face During the Dyadic Bid

Data from all 124 participants was entered into the analysis. A Kruskal–Wallis H test indicated no outcome group difference in proportional dwell time on the actress’ face during the dyadic bid preceding the gaze shifts [*H*(3) = 0.196, *p* = 0.978]. Median face dwell times were high for all outcome groups: LR (Mdn = 0.909, range = 0.527 to 1); HR-TYP (Mdn = 0.942, range = 0.034 to 1); HR-ATYP (Mdn = 0.940, range = 0.309 to 1); and HR-ASD (Mdn = 0.931, range = 0.394 to 1) indicating that all groups engaged with the actress while she was addressing them.

### Gaze Following: First Look Direction to Referent vs. Distractor

Only participants with two or more trials starting on the face and with at least one first look to an object entered the analysis directly comparing looks to the referent and the distractor (100 participants: 21 LR, 48 HR-TYP, 22 HR-ATYP, 9 HR-ASD). There were no outcome group differences in the number of trials in which gaze started on the face at the beginning of the gaze shift either before or after exclusion criteria were applied, with all groups contributing approximately five trials to this analysis. Comparisons to chance were completed using the non-parametric Wilcoxon signed-rank test. All outcome groups followed gaze to the referent in most trials, which led to performance of all groups being significantly above chance level (0): LR (Mdn = 1.0, range = −1 to 1), *z* = 3.829, *p* < 0.001; HR-TYP (Mdn = 1.0, range = −1 to 1), *z* = 5.735, *p* < 0.001; HR -ATYP (Mdn = 1.0, range = −1 to 1), *z* = 4.164, *p* < 0.001; and HR-ASD (Mdn = 1.0, range = 0.333 to 1), *z* = 2.887, *p* = 0.004. A non-parametric Kruskal–Wallis H test indicated a trend toward significant difference between outcome groups [*H*(3) = 7.712, *p* = 0.052]. However, pairwise comparisons with adjusted *p*-values were not significant (all *p* > 0.90, except HR-TYP vs. HR-ASD, *p* = 0.451; HR-TYP vs. HR-ATYP, *p* = 0.095).

### Gaze Following: Dwell Time to Referent vs. Distractor

**FIGURE 2:**
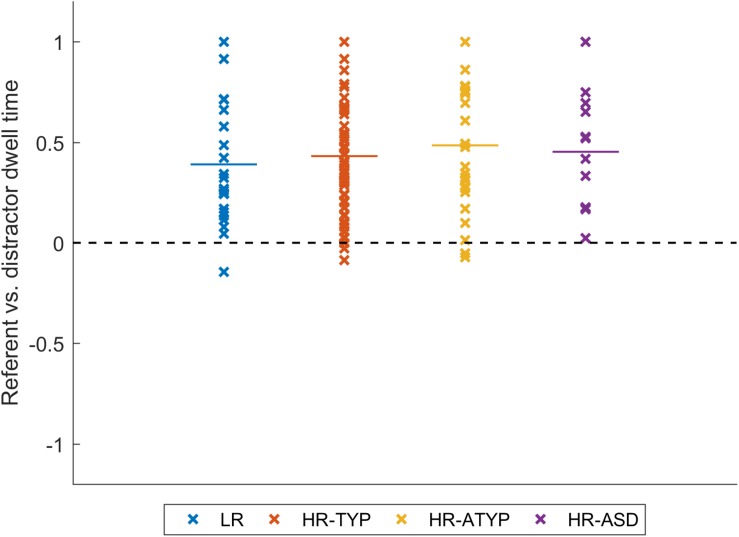
Gaze following: dwell time to referent vs. distractor.

Data from 123 participants entered the analysis (1 HR-TYP infant never looked at either referent or distractor); number of participants differed from the previous analysis since we also included trials in which the infant did not start on the face (see section “Data Reduction”). An analysis of covariance using outcome as the between participant factor, age and data quality as covariates and number of trials contributing as a weighting factor, indicated no significant differences between outcome groups, *F*(3,117) = 0.391, *p* = 0.760; see Figure 2. Covariate effects were also non-significant (age, *p* = 0.225; data quality, *p* = 0.379). All outcome groups showed significantly greater than chance preference for the referent over the distractor [LR, *M* = 0.390, *SD* = 0.316, *t*(22) = 5.916, *p* < 0.001; HR-TYP, *M* = 0.433, *SD* = 0.291, *t*(61) = 11.735, *p* < 0.001; HR-ATYP, *M* = 0.483, *SD* = 0.329, *t*(25) = 7.494, *p* < 0.001; HR-ASD, *M* = 0.453, *SD* = 0.291, *t*(11) = 5.388, *p* < 0.001].

### Attention Distribution: Dwell Time to All Areas of the Screen

**FIGURE 3:**
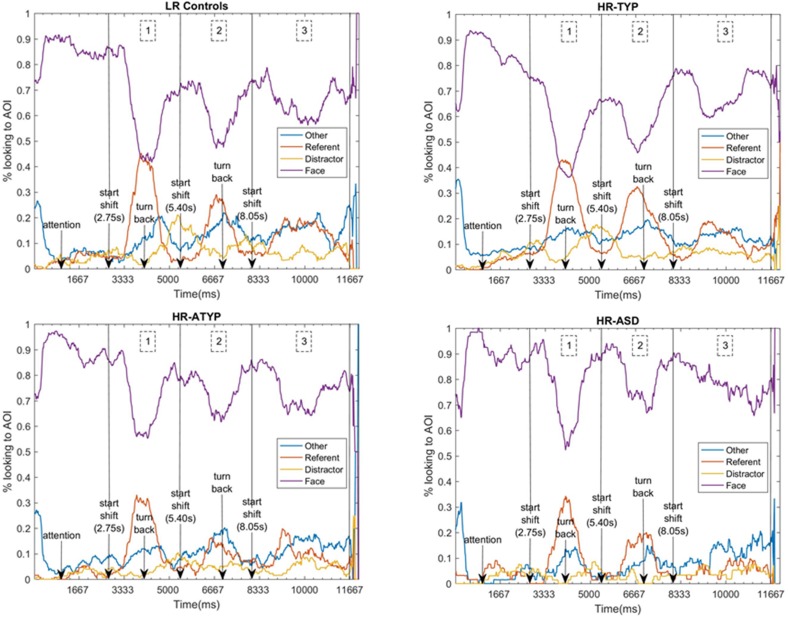
Time-course of attention to AOIs for each outcome group, indicating events and the three gaze shift time-segments analyzed. 1 denotes the period of the first gaze shift, shift 1 (2750–5400 ms); 2 the second gaze shift, shift 2 (5400–8050 ms); and 3 the third gaze shift, shift 3 (8050–11670 ms).

**FIGURE 4:**
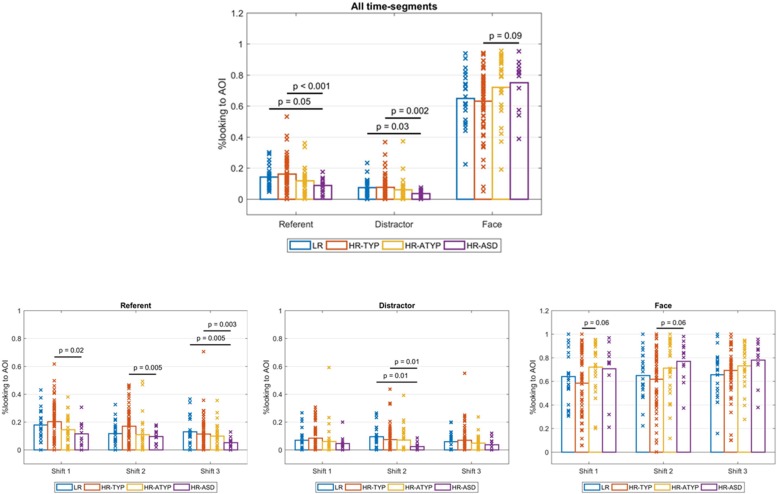
Outcome group comparisons: proportions looking to AOIs by AOI across all shift time-segments and by AOI for each shift time-segment.

Data from all 124 participants entered the analysis. Figure 3 shows the time course of attention distribution for the four outcome groups. A main effect of AOI was found [Wald χ^2^(3) = 1247.832, *p* < 0.001] with proportional dwell time on the face significantly greater and dwell time on distractor significantly reduced compared to other AOIs; dwell time on referent and other areas of the screen were not significantly different. No main effects of outcome [Wald χ^2^(3) = 0.471, *p* = 0.925] or time-segment were found [Wald χ^2^(2) = 0.012, *p* = 0.994]. A significant outcome group × AOI × time-segment interaction was found [Wald χ^2^(18) = 43.949, *p* = 0.001; see Figure 4]. This was followed-up with 4 GEEs, one for each AOI (referent, distractor, face and other parts of the screen) which are described in the following sections.

#### Attention to the Referent Relative to Screen Time

For dwell time on the referent there was a significant main effect of outcome [Wald χ^2^(3) = 18.744, *p* < 0.001]; see Figure 4. Bonferroni corrected pairwise comparisons indicated HR-ASD looked at the referent less than LR controls (marginally significant at *p* = 0.054) and HR-TYP (*p* < 0.001). There was a main effect of time-segment [Wald χ^2^(2) = 29.152, *p* < 0.001]. Bonferroni corrected pairwise comparisons indicated looking to the referent decreased significantly from first to second and second to third gaze shifts (all *p*s < 0.05). The outcome × time-segment interaction was significant [Wald χ^2^(6) = 13.065, *p* = 0.042]. The model was re-run for each time-segment to break down the interaction effect. Pairwise comparisons were run with Bonferroni correction. There were significant differences between outcome groups during the first [Wald χ^2^(3) = 10.447, *p* = 0.015] second [Wald χ^2^(3) = 11.909, *p* = 0.008] and third gaze shift [Wald χ^2^(3) = 18.199, *p* < 0.001]; see Figure 4. In the first two gaze shifts HR-ASD looked at the referent significantly less than HR-TYP (first, *p* = 0.021, second, *p* = 0.005). In the third gaze shift HR-ASD looked at the referent significantly less than LR (*p* = 0.005) and HR-TYP (*p* = 0.003).

#### Attention to the Distractor Relative to Screen Time

For dwell time on the distractor there was a significant main effect of outcome [Wald χ^2^(3) = 17.346, *p* = 0.001]; see Figure 4. Bonferroni corrected pairwise comparisons indicated HR-ASD looked at the distractor significantly less than LR (*p* = 0.033) and HR-TYP (*p* = 0.002). There was no main effect of time-segment (Wald χ^2^(2) = 3.124, *p* = 0.210) and the outcome × time-segment interaction was not significant [Wald χ^2^(6) = 9.721, *p* = 0.137].

#### Attention to Background (i.e., Outside the Main AOIs) Relative to Screen Time

For dwell time on areas other than the face, referent or distractor there was no main effect of outcome [Wald χ^2^(3) = 1.873, *p* = 0.599], time-segment [Wald χ^2^(2) = 2.200, *p* = 0.333] or outcome × time-segment interaction [Wald χ^2^(6) = 7.828, *p* = 0.251].

#### Attention to the Actress’ Face Relative to Screen Time

There was a significant main effect of outcome [Wald χ^2^(3) = 8.235, *p* = 0.041]. However, with Bonferroni correction no significant pairwise differences were found, although difference between HR-ASD and HR-TYP showed a trend (*p* = 0.086), with HR-ASD looking longer to faces. There was a significant main effect of time-segment [Wald χ^2^(2) = 6.764, *p* = 0.034]. Bonferroni corrected pairwise comparisons indicated looking to the face increased significantly from first to third gaze shifts (*p* = 0.028) but not from first to second (*p* = 0.248) or second to third gaze shifts (*p* = 0.145). The outcome × time-segment interaction was not significant [Wald χ^2^(6) = 11.483, *p* = 0.075].

When the treatment variable, indicating those who took part in a parent-mediated intervention, was included in the model the main effect of outcome became marginally significant [Wald χ^2^(3) = 7.612, *p* = 0.055]; main effects of time-segment [Wald χ^2^(2) = 6.789, *p* = 0.034] and outcome × time-segment interaction remained unchanged [Wald χ^2^(6) = 11.480, *p* = 0.075].

#### Does Looking Longer at Faces Associate With Better Gaze Following?

To investigate whether the amount of time looking at the face during gaze shifts impacts gaze following abilities, we employed the gaze following measures directly comparing referent and distractor (ref − dist)/(ref + dist) both for first look direction (not normally distributed so using Kendall’s tau) and for dwell time. We found that face dwell time during gaze shifts did not associate with first look direction either for the whole sample (*τ*b = 0.099, *n* = 103, *p* = 0.197) or for the HR siblings only (*τ*b = 0.092, *n* = 82, *p* = 0.287) but *did* positively associate with relative dwell time both for the whole sample (*r* = 0.400, *n* = 123, *p* < 0.001) and the HR siblings only (*r* = 0.424, *n* = 100, *p* < 0.001).

### Correlations Between Dwell Times and Phenotypic Measures

There were no significant correlations between the gaze following dwell time measure (referent vs. distractor) and phenotypic measures. Neither were there associations between attention distribution dwell time measures and ASD symptoms. However, there were significant associations between attention distribution dwell times and both concurrent and later language measures (Table 3). In summary, both referent and distractor dwell times positively correlate with concurrent and later verbal and composite measures while negative correlations are found for face dwell time. The opposite direction of these associations is expected from the fact that face and object dwell times are also correlated. Only a subset of the associations, predominantly with measures of language development, survive corrections for multiple comparisons: concurrent CDI associates positively with referent dwell time, while 36-month verbal MSEL associated positively with both referent and distractor dwell times and negatively with face dwell time. Supplementary Material reports the full set of correlations run for attention distribution measures with the high-risk only group, which follows a similar pattern to the whole cohort (S5), and associations found between first look and phenotypic measures (S6).

**TABLE 3:** Associations between attention distribution during the teaching trials and phenotypic measures.

		**Dwell time Referent vs. Distractor**	**Dwell time, referent**	**Dwell time, distractor**	**Dwell time, face**
ADI 36 mo.	Socia	0.055	–0.059	–0.066	0.050
	Comm	0.034	–0.153	–0.108	0.112
	RR	0.063	–0.050	–0.072	0.044
ADOS 36 mo.	Social affect	–0.034	–0.092	–0.041	0.094
	RRB	0.095	–0.072	–0.130	0.095
SRS 36 mo.	t-score	0.076	–0.055	–0.048	0.014
CDI words understood	15 mo.	–0.031	**0.188^∗^**	0.141	−0.170^∗^
	24 mo.	−0.075	0.177	0.111	−0.138
MSEL verbal	15 mo.	0.058	0.247^∗^	0.134	−0.205
	36 mo.	−0.128	**0.262^∗^**	**0.205^∗^**	−**0.272^∗^**
MSEL Non-verbal	15 mo.	−0.080	0.143	0.139	−0.178
	36 mo.	−0.151	0.172	0.153	−0.251^∗^
MSEL ELC (total)	15 mo.	0.006	0.240^∗^	0.143	−0.228^∗^
	36 mo.	−0.155	0.229^∗^	0.192^∗^	−**0.281^∗^**
MSEL ELC (total)	36 mo.^$^	−0.167	0.117	0.121	−0.214

*ADI (The Autism Diagnostic Interview – Revised); ADOS (The Autism Diagnostic Observation Schedule), values used were calibrated severity scores; SRS (Social Responsiveness Scale), value was the total t-Score; CDI words understood (MacArthur-Bates Communicative Development Inventory) was derived by summing words understood only and words understood and spoken; MSEL (Mullen Scales of Early Learning), MSEL Verbal combined verbal subscales (receptive and expressive language), MSEL Non-Verbal combined two non-verbal subscales (visual reception and fine motor), MSEL ELC (Early Learning Composite) combined those four subscales. Where both values were normally distributed, parametric Pearson’s r is reported (shaded) otherwise where one or both variables were not normally distributed, values are Kendall’s tau (unshaded). All values are uncorrected for multiple comparisons. ^∗^*p* < = 0.01 level; bold values indicate where correlations remained significant after correction for multiple comparison (*p* < = 0.004). ^$^Accounting for 15 mo. MSEL.*

## Discussion

This study asked whether atypicality in gaze following in infants later diagnosed with ASD (HR-ASD) reported in previous literature reflects poor understanding of gaze direction or differences in attention distribution to the visual scene and whether these putative differences lead to poorer object-label mapping by this group. We also tested whether the ability to follow gaze or to optimally distribute attention when learning words associates with later clinical and developmental outcomes. Our main findings were:

(1) As predicted, HR-ASD infants had intact gaze following as measured by direct comparison of first look direction. HR-ASD infants did not differ from other outcome groups with all groups directing significantly more first looks to the referent than the distractor.
(2) In agreement with Bedford et al. (2012), HR-ASD infants engaged significantly less with referents than HR-TYP and LR infants (i.e., shorter dwell times to the referent measured as a proportion of screen looking). However, HR-ASD infants also looked at distractors significantly less than typically-developing outcome groups. Thus, when dwell times to referent and distractor were compared directly, no outcome group differences were found.
(3) Since we found no evidence of object-label mapping (‘word learning’) for any outcome group the task appears to have been too challenging for this age group. However, infants’ attention distribution measures from teaching trials associated with concurrent and later language.
(4) We were unable to make a clear prediction regarding dwell time on the face but previous literature suggested that HR-ASD infants would spend less or equal amount of time on faces when compared with typically developing groups. We found no outcome group differences in dwell time on the face either during the dyadic bid or whilst the actress was making gaze shifts.

We discuss the main findings in more detail further on. We begin by discussing engagement with the actress’ face since this is an important precursor and on-going aid to successful use of gaze information.

### Engaging With the Face

HR-ASD infants engaged similarly with the face prior to and during the actress’ gaze shifts. This is contrary to previous studies reporting less visual attention to faces (Chawarska et al., 2013; Jones and Klin, 2013). However, two of three studies which analyzed face dwell time in gaze following paradigms, also failed to find less looking to the face (Chawarska et al., 2012; Billeci et al., 2016). Chawarska et al. (2012) suggested decreased face dwell time may occur specifically during dyadic bids, especially when there are long periods of direct gaze and explicit cues for engagement. This is supported by the studies listed in Table 1.1 in which differences were found when actors posed questions and/or entreated infants to join in with actions (Jones et al., 2008; Chawarska et al., 2012, 2013; Jones and Klin, 2013; Nyström et al., 2017). Contrastingly, in gaze following paradigms like ours, direct gaze is necessarily sporadic and speech, when included, mainly consists of greetings and a brief narrative. In this context, where fewer demands are made for infant response, those with ASD or later ASD may be less inclined to shift attention away from the face.

A particular characteristic of our stimuli may have held HR-ASD infants’ attention on face. The repeated gaze shift in these clips meant that the face was frequently in motion. Some studies have suggested that perceptual salience, driven by movement or luminance contrast, may be more influential in the visual attention of young children with ASD (e.g., Amso et al., 2014) or infants with later ASD (e.g., Cheung et al., 2018; Nyström et al., 2018). Follow-up analysis of the association between longer face looking and subsequent better differential engagement with the referred object suggested that looking longer at faces, as they repeatedly turned toward one of the objects, may have a positive impact on the use of gaze cues. However, since longer looking toward faces does not predict better direction of first looks, this seems to suggest that it does not necessarily benefit infant’s reading of gaze direction. Given most children directed their first look to the referent, looking to faces for longer may simply have not left them enough time to also look at the distractor.

### Intact Gaze Following

In common with previous similar screen-based eye-tracking studies with younger infants (see Table 1.2), results suggested that gaze following, operationalized as more first looks or dwell time directed to the referent compared to the distractor, is intact in HR-ASD infants. It remains unclear what the mechanisms are that allow infants to shift attention in the direction of someone’s gaze shift. The head turn used in this and many other gaze following paradigms may entice an infant to follow because they understand and act upon the actor’s communicative intent, or because head movement acts as an exogenous cue which sets the infant’s gaze in the congruent direction. Gaze following in early infancy appears exogeneous, occurring even when an actor’s eyes are closed but typically-developing infants begin to understand the referential nature of another’s gaze in the second year (e.g., Corkum and Moore, 1995; Caron et al., 2002; Brooks and Meltzoff, 2005). Thus, both mechanisms begin to act but even though infants might understand referential intent, exogenous cues remain influential. For example, a recent study with typically-developing 12-month-olds suggested infants’ attention in joint play may be more attributable to exogenous cues present in the interaction, such as objects being held and moved, than to endogenous control from the infant (Wass et al., 2018). If gaze following measured by first look direction were primarily exogenously driven, intact gaze following in infants later diagnosed with ASD would be unsurprising since exogenously cued attention orienting has been shown to be typical in this population (e.g., Elsabbagh et al., 2013). However, we also found that all groups engaged more with the referent than the distractor and there were no outcome group differences in how attention was distributed between referent and distractor. Hence this does not support the hypothesis of poorer understanding of the referential meaning of gaze in HR-ASD. Nevertheless, we discuss below whether this conclusion can be generalized to all settings in which gaze following has been measured.

### Atypical Distribution of Attention

When considering attention distribution to the whole screen (i.e., referent vs. distractor vs. face vs. background), HR-ASD infants looked less at both referent and distractor when compared to HR-TYP and LR infants. Billeci et al. (2016) also found toddlers with ASD spent less time looking at a distractor object, making more transitions back and forth from referent to the face whereas typically-developing toddlers made more transitions back and forth between the two objects.

What could explain less looking at objects in HR-ASD infants? Our proportional measure does not allow us to tell whether this difference is driven by looking more toward faces and other areas of the screen or less toward objects. We thank a reviewer for suggesting that we look at whether HR-ASD also engage less with objects during the test trials when the actress was not present. If that were the case it may suggest that HR-ASD are unable or unwilling to engage with objects rather than failing to do so because they looked too long at faces during the teaching phase. However, a Kruskal–Wallis H tests found no evidence that looking to objects (as a proportion of screen time) during the test trials differed by outcome group, *H*(3) = 1.255, *p* = 0.740. There were no differences when looking either during the baseline, *H*(3) = 0.493, *p* = 0.920 nor while the objects were labeled, *H*(3) = 4.564, *p* = 0.207. Thus, lesser looking at objects may be a result, in part, from longer looking toward the face.

Importantly, it was the dwell times to individual AOIs and not the distribution of attention between referent and distractor that showed associations with concurrent and later verbal development and vocabulary. This suggests that the amount of time spent engaged with objects may be more important for language acquisition than understanding and following gaze *per se*. This is an intriguing finding in the autism literature, which has often given prominence to gaze following difficulties as a key limitation to language acquisition, but it accords with some recent findings from studies of both typical development and children with ASD. Yu et al. (2018) showed that 9-month-olds’ amount of sustained attention to objects during naming episodes was a stronger predictor of vocabulary a few months later than the amount of time infant and parent spent jointly attending to object. This is because parents often choose to label objects the infant is already attending to, thus relieving them from the need to follow gaze to discover the referent of uttered words (Yu and Smith, 2013). In support of this hypothesis, Adamson et al. (2017), investigating joint attention in toddlers with ASD, found that the amount of time spent jointly engaged with objects but not the amount of time in which toddlers shifted attention between objects and parent, associated with later expressive vocabulary. Engaging with objects for longer while infants receive information about these objects (e.g., labels) probably increases the opportunity to encode both objects features and the object-label association to memory.

### The Validity of Screen-Based Measures of Joint Attention

While recent findings from naturalistic parent child interaction (Yu and Smith, 2013; Wass et al., 2018) cast doubt on the validity of screen-based measures of joint attention, the fact that ours associated with later language measures supports the contention that screen-based measures can and do capture important differences in the dynamics of visual attention that are relevant beyond the experimental settings in which they are measured.

However, there is a sense that screen-based interaction may not challenge children with ASD as much as live interaction, thus underestimating the severity of their difficulties with real-world gaze following. Few studies have measured gaze following in live interaction in infants with later ASD using eye-tracking. As in our study, Nyström et al. (2019), found no outcome difference in the amount of first looks directed to referent vs. distractor in 10-month olds infants at risk for ASD. In contrast, Presmanes et al. (2007) and Sullivan et al. (2007) showed that HR-ASD infants directed fewer first looks to referents but they did not employ eye tracking which means we do not know where infants look when they did not correctly follow gaze, i.e., did they make an incorrect first look to another object or did they not disengage from the face? To clarify these differences, rather than making a distinction between live or screen-based studies, future studies should more carefully characterize the experimental variables that may differentiate these settings. For example, it may be that live settings are also more cluttered, presenting more opportunity for distracting attention from the task. However, it is notable that attention distribution was atypical in HR-ASD even in our sparse visual scenes.

## Conclusion

We set out in the introduction different reasons why infants who are later diagnosed with ASD may not use referential cues, such as gaze direction, appropriately. We show that, in our paradigm, this is not because these infants fail to engage with faces. As in Bedford et al. (2012), we show that all groups make a correct first look to the referent object, compared to a distractor, but that those infants who go on to develop ASD spend proportionally less time on the referent object (scaled to screen looking) compared to low risk controls and high-risk infants that go on to typical outcomes. However, in contrast to Bedford et al. (2012), we also explored attention distribution to other areas of the screen which revealed that infants with later ASD did not spend less time on the referent compared to the distractor but spent less time engaged with objects overall. Our findings therefore support the idea that in controlled communicative contexts, triangulating gaze direction and understanding the referential content of gaze shifts are typical during the early development of infants with later ASD, but that attention is not distributed optimally. Although all the above measures have been used in the literature to index gaze following, the current study highlights key differences in terms of the underlying processes they capture and emphasizes the importance of taking this into consideration when choosing how to operationalize this complex behavior. Beyond this methodological point, we also offer an interpretation of emerging differences in attention distribution. We suggest that processing differences, reflecting either a bias to salient features such as movement or difficulties in extracting information from the face and gaze interfere with the optimal distribution of attention, in particular with engaging with relevant information in joint attention scenarios (i.e., with the objects infants have to learn about). Thus, although differences in attention distribution do not selectively map onto later ASD traits, they are a marker of developmental delay or atypicality and a potential predictor of later language abilities which means that they could become an important stratification dimension.

## Data Availability

The datasets generated for this study are available on request to the corresponding author.

## Ethics Statement

This study was carried out in accordance with the recommendations of the NHS National Research Ethics Service (NHS RES London REC 08/H0718/76; 14/LO/0170). Parental written informed consent was obtained for all participants in the study in accordance with the Declaration of Helsinki.

## Author Contributions

TG, RB, TC, and MJ designed the study. JP, TG, RB, and EJ analyzed the data. All authors contributed to writing up the study.

## Conflict of Interest Statement

The authors declare that the research was conducted in the absence of any commercial or financial relationships that could be construed as a potential conflict of interest.
