# Supplementary material for: Gaze Following and Attention to Objects in Infants at Familial Risk for ASD
Source: Front Psychol. 2019 Aug 20;10:1799. doi: 10.3389/fpsyg.2019.01799 (PMC6710391; doi:10.3389/fpsyg.2019.01799)
Supplement: Supplementary file 1 [file Data_Sheet_1.docx]

**S1. Older siblings of participants**

For the 101 participants contributing data to this study, each of whom had an older sibling with a community clinical diagnosis of ASD (hereafter probands), parents completed the Development and Well-Being Assessment (DAWBA; Goodman, Ford, Richards, Gatward & Meltzer, 2000) and/or the Social Communication Questionnaire (SCQ; Rutter, Baily & Lord, 2003) for the probands.  Eighty probands met criteria on both the DAWBA and SCQ.  While a small number scored below threshold on the SCQ (n = 7), no exclusions were made due to meeting threshold on the DAWBA and expert opinion.  For 13 probands, confirmation of local clinical diagnosis was only available for the SCQ.

Screening for possible ASD in the older siblings of the low risk (LR) infants was undertaken using the SCQ, with no child scoring above the instrument cut-off for ASD (>15). Medical history review confirmed an absence of ASD within first-degree relatives.

**S2. Test trials and learning**

*S2.1 Data reduction and analytic approach*

Successful object-word mapping was defined as an increase in looking towards the referent after word labels were spoken (labelling phase) compared to before (baseline phase). Two levels of difficulty were presented. In one-word trials infants need only remember to which object the person referred, in two-word trials infants had to map each word to the corresponding object. We defined the labelling phase as starting 300ms after the offset of the first label (as in Fernald, Marchman and Weisleder, 2013) which is believed to give infants enough time to process the word. The baseline phase preceded this time point and started 300ms into the test trial. For one-word trials the baseline phase was 300-2783ms and labelling phase was 2800-7500ms. For two-word trials the baseline phase was 300-3033ms and labelling phase 3050-7500ms. Trials were included where accumulated looking time to objects exceeded 300ms in both phases and, during the baseline time-segment, infants must have looked at both referent and distractor. Object-word mapping in both phases was measured by the dwell time on the referent scaled by dwell time on either object: R/(R+D). This was the measure used by Gliga et al. (2012) when testing whether infants could learn from gaze following and it also follows the approach taken to ‘looking while listening’ measurements by Fernald, Marchman and Weisleder. Values range from 0, where dwell time is directed exclusively to the distractor, to +1 where dwell time is directed exclusively to the referent. The chance level is 0.5.

There were 105 participants who had at least one valid trial from either one or two-word tests (21 LR, 53 HR-TYP, 20 HR-ATYP, 11 HR-ASD). In order to include participants who did not provide data for both trial types and, because baseline and labelling phases are likely to be correlated for a given participant, we used a Generalized Estimating Equation (GEE) approach. The object-word mapping measure was entered into a Gaussian model with identity link (participant id) between predictors and expected proportions. An unstructured working correlation matrix was chosen. Trial type (one or two word) and phase (baseline or labelling) were added as within-participant factors and outcome as a between-participants factor. Data quality and age in months at the time of the study were added as covariates and number of trials contributing was used as a weighting factor.

*S2.2 Object -label mapping results: referent scaled by object dwell time*

There were no differences between outcome groups in trials contributing (p = .638), age (p = .830), or data quality (p = .318). There were no significant main effects of outcome [Waldχ^2^ (3) = 5.884, p = .117], trial type [Waldχ^2^ (1) = .005, p = .943] or phase [Waldχ^2^ (1) = .625, p = .429]. The outcome x phase interaction was significant [Wald χ^2^(3) = 11.272, p = .010], but no other interactions were significant suggesting there were no differences in object-label mapping between one and two-word trials; see **Figure S1**. The model was re-run for each phase to break down the interaction effect. In the baseline phase there were no differences between outcome groups [Wald χ^2^(3) = .899, p = .828]. However, in the labelling phase there were significant outcome group differences [Wald χ^2^(3) = 12.141, p = .007]. Bonferroni corrected pairwise comparisons indicated LR looked less at the referent as a proportion of object dwell time than HR-TYP infants (p = .005).

Chance comparisons were completed using one-sample t-tests. Outcome groups showed no difference from chance (0.5) during the baseline [LR, M = .504, SD= .165, t(37) = .143, p = .887 ; HR-TYP, M = .502, SD = .149, t(94) = .145, p = .885; HR-ATYP, M = .480, SD = .139, t(34) = -.860. p = .396; HR-ASD, M = .475, SD = .129, t(18) = -.872, p = .394]. However, during the labelling phase, LR controls differed significantly from chance (0.5), showing less looking to the referent as a proportion of object dwell time [LR, M = .392, SD = .246, t(37) = -2.701, p = .010]. Other outcome groups showed no difference from chance [HR-TYP, M = .496, SD = .246, t(94) = -.178, p = .859; HR-ATYP, M = .467, SD = .281, t(34) = -.698, p = .490; HR-ASD, M = .486, SD = .278, t(19) = -.226, p = .823].

When the treatment variable, indicating those who took part in a parent-mediated intervention, was included in the Generalized Estimating Equation model the main effect of outcome became significant [Waldχ^2^ (3) = 8.512, p = .037]. Bonferroni corrected pairwise comparisons indicated LR controls looked significantly less at the referent as a proportion of object dwell time than HR-TYP infants (p = .027). Main effects of trial type [Waldχ^2^ (1) = .004, p = .950] and phase remained unchanged [Waldχ^2^ (1) = .623, p = .430] and the outcome x phase [Wald χ^2^(3) = 11.259, p = .010] continued to be the only significant interaction.

| 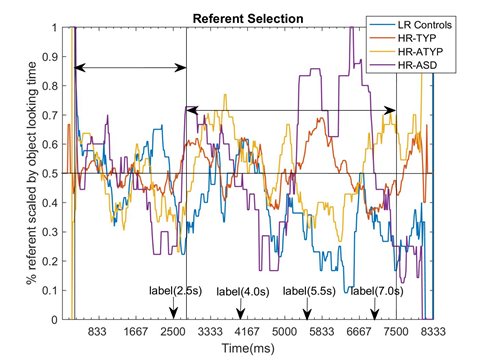 | 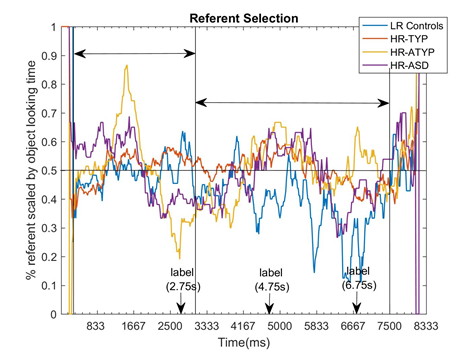 |
| --- | --- |
| 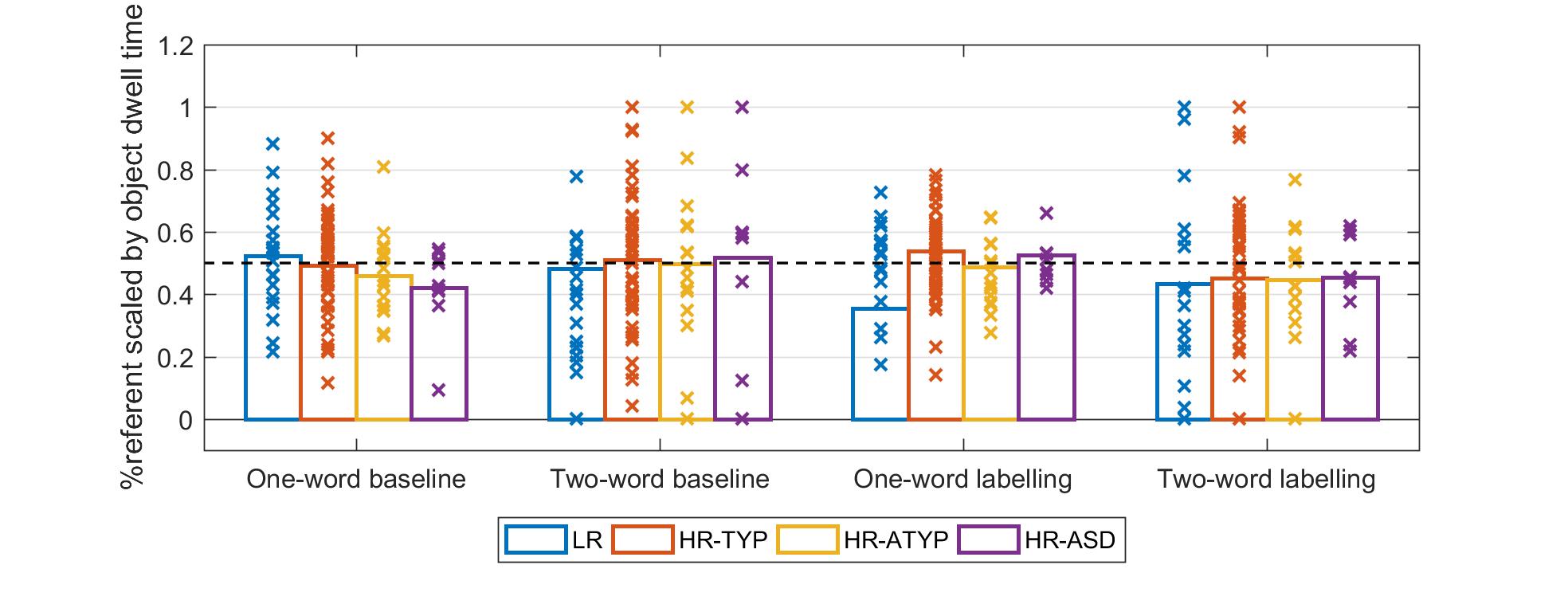 | |

**Figure S1:** ***Top:*** Time-course of attention for outcome groups for one-word (on left) and two-word (on right) test trials indicating phases (baseline, 300ms to first label plus 300ms; labelling, first label plus 300ms to end); ***Bottom:*** Outcome group comparison of referent scaled by object dwell time by trial type and phase.

*S2.3 Object -label mapping results: longest look difference score*

It has been suggested that the difference in longest looks may be more accurate than overall dwell times in assessing learning (Schafer & Plunkett, 1998). We therefore repeated analyses with this measure. Difference in longest look was derived by subtracting the longest look to the distractor from that to the referent and scaling with the sum of both (Longest Look_referent_ – Longest Look_distractor_ / (Longest Look_referent_ + LongestLook_distractor_). Trials were excluded as previously. An average was calculated across valid trials for each participant for each phase and trial type.

Using an identical Generalized Estimating Equation approach with the same 105 participants entering the analysis, we found no significant main effects of outcome [Waldχ^2^ (3) = 4.389, p = .222], trial type [Waldχ^2^ (1) = .753, p = .385], phase [Waldχ^2^ (1) = .344, p = .558] nor any significant interactions. Addition of the treatment variable to the model did not change significance for main effects or interactions.

*S2.4 Discussion*

We found no evidence of object-label mapping (‘word learning’) in any outcome group either when analyzing dwell times or longest looks. This may be because there were not enough repetitions of the word during teaching; we used 6 repetitions across 2 trials whereas 9 repetitions were needed for typically-developing 13-month-olds to succeed in a face-to-face 5-minute training session (Woodward Markman & Fitzsimmons, 1994). Furthermore, evidence with typically-developing 12 to 14-month-olds using screen-based paradigms like ours is mixed. Successful object-label mapping has been reported with 20 repetitions (Paulus & Fikkert, 2014) whilst unsuccessful object-label mapping has been found with more repetitions than in our study (Briganti and Cohen, 2011, 18 repetitions; Yorovsky & Frank, 2014, 12 repetitions). Some have argued that typically-developing infants under 19-24 months may notice attentional cues offered by a speaker but may be unable to use this information consistently to make object-label associations because they lack the ability to take the speaker’s point of view and ignore perceptual salience (Parish-Morris et al, 2007). These results would support such inconsistency; the ability to map labels to objects may be fragile at 14-15 months requiring significant support from repetition and perhaps face-to-face, more naturalistic interaction to succeed. However, at this age infants can readily learn words in screen-based paradigms when objects are presented in isolation and labelled in voiceover (Pomiechowska & Gliga, 2019; Yin & Csibra, 2015). Recent studies employing head-mounted cameras and eye-tracking which observed parents and infants in interaction, suggested that most successful labelling events occurred when the object filled a great proportion of infants’ visual field (Yu & Smith, 2016), as is the case in Pomiechowska & Gliga. When in close proximity to parents, infants also spend little time on the parent’s face and a great proportion of time on objects and hands (Yu & Smith, 2013; Wass et al., 2018). Thus, it may be that screen-based paradigms like ours do not best capture the real-life dynamics of word learning.

Finally, in the dwell time analysis we found that the LR group showed not just chance performance in the labelling phase during one-word trials but an unexpected less than chance preference for the referent, thus greater preference for the distractor. This may have arisen because the location of the distractor in both one-word test trials corresponded to the location of the referent object in the immediately preceding teaching trial. Thus, on hearing the label LR infants may just have been returning to that location. However, there is evidence that typically-developing infants use space to link objects and labels (Samuelson, Smith, Perry & Spencer, 2011), hence this spatial memory may have overridden the social teaching cue leading to incorrect object-label mapping in the LR group. As far as we are aware, no research has explored whether the influence of spatial memory on object-label mapping differs in infants with familial ASD risk and, as there was a significant difference between the LR and the HR-TYP group (who were at chance), this may be an interesting line of future research.

**S3. A closer replication of Bedford et al., 2012: First look distribution**

We analyzed the broader distribution of first looks to referent, distractor, the face and the background, scaled by the number of valid first looks (see also Bedford et al., 2012). Since first look directions will be correlated, a Generalized Estimating Equation (GEE) approach with an unstructured working correlation matrix was chosen. The analysis used a Gaussian model with identity link (participant id) between predictors and expected proportions and with AOI as a within-participant and outcome group as a between-participant variable.

When comparing first look distributions to all AOIs (see **Table S1**), we only included participants with two or more trials starting on the face (109 participants: 22 LR, 52 HR-TYP, 24 HR-ATYP, 11 ASD). After exclusion criteria were applied, there were no outcome group differences in age (p = .874), number of trials starting on the face (p = .621) or data quality (p = .319). There was no significant main effect of outcome [Waldχ^2^ (3) = .152, p = .985] but there was a significant main effect of AOI [Waldχ^2^ (3) = 718.913, p < .001]. Staying on the face and first look direction to referents were significantly more frequent than first looks to distractors or other areas of the screen (all ps <.001). First looks to distractors were significantly less frequent than to other areas of the screen (p < .001). There was no significant difference between staying on the face and first looks to referents (p = .342).

A significant outcome x AOI interaction was found, Waldχ^2^ (9) = 22.666, p = .007. There were no significant outcome group differences for first looks to the referent [Waldχ^2^ (3) = 1.927, p = .588], staying on the face [Waldχ^2^ (3) = 5.616, p = .132] or to other areas of the screen [Waldχ^2^ (3) = 5.233, p = .155]. However, significant outcome group differences were found for the distractor [Waldχ^2^ (3) = 14.789, p = .002] with HR-TYP directing more first looks to the distractor than atypical groups (HR-ATYP, p = .003; HR-ASD, p = .006).

To understand this unexpected result, and since objects presented as referents reappeared as distractors in the second half of the experiment, we performed a post-hoc test to determine whether HR-TYP infants directed more first looks to the distractor in the second four trials than the other outcome groups. After adding a term to denote the first or second four trials, with the same participants (22 LR, 52 HR-TYP, 24 HR-ATYP, 11 ASD) entering the analysis, we found no main effects for outcome [Waldχ^2^ (3) = .131, p = .988], trial type [Waldχ^2^ (1) = .683, p = .408] or age as a covariate [Waldχ^2^ (1) = .767, p = .381]. There was a significant main effect of AOI as before [Waldχ^2^ (3) = 1742.509, p < .001]. However, we also found a significant outcome x AOI x trial-type interaction, Waldχ^2^ (9) = 17.274, p = .045.

Following up for each trial type, the outcome x AOI interaction remained significant only for the second four trials, Waldχ^2^ (8) = 32.421, p < .001 (105 participants: 22 LR, 49 HR-TYP, 23 HR-ATYP, 11 ASD). Breaking down the interaction by AOI, there was only a significant difference between outcome groups for first looks to the distractor [Waldχ^2^ (3) = 20.179, p < .001], which with pairwise correction, indicated that, in the second four trials only, HR-TYP infants directed significantly more first looks to the distractor than any other outcome group (LR, p = .020; HR-ATYP, p < .001; HR-ASD, p < .001). This suggests the HR-TYP group may have directed their first looks to the distractor in the second part of the study, because they remembered these objects as having been referents in previous trials.

|  | | | **HR-ASD** | **HR-ATYP** | **HR-TYP** | **LR Controls** | **p** |
| --- | --- | --- | --- | --- | --- | --- | --- |
| **Trials starting on the face^a^** | | |  |  |  |  |  |
| N  Mean (SD)  Median  Proportion of trials with data | | | 11  5.91 (1.921)  7.00  .779 | 24  5.50 (1.888)  5.50  .748 | 52  5.29 (1.934)  5.00  .723 | 22  5.09 (1.743)  5.00  .695 | .621  .781 |
| **Out of those trials** | | |  |  |  |  |  |
|  | **Gaze staying on face** | |  |  |  |  |  |
|  | | Mean (SD) Proportion of trials^b^ | 2.18 (1.779)  .369 | 2.29 (1.781)  .417 | 1.38 (1.682)  .262 | 1.82 (2.152)  .357 | .132 |
|  | **First look to referent** | |  |  |  |  |  |
|  | | Mean (SD) Proportion of trials^b^ | 2.55 (2.162)  .432 | 2.33 (1.736) .426 | 2.67 (1.581) .506 | 2.64 (1.761) .517 | .588 |
|  | **First look to distractor** | |  |  |  |  |  |
|  | | Mean (SD) Proportion of trials^b^ | .09 (.302)  .015^c^ | .08 (.282)  .014^c^ | .44 (.669)  .083^c^ | .23 (.528)  .045 | **.002** |
|  | **First look to other areas** | |  |  |  |  |  |
|  | | Mean (SD) Proportion of trials^b^ | 1.09 (1.044)  .184 | .79 (1.021)  .143 | .79 (1.016)  .149 | .41 (.590)  .081 | .155 |

**Table S1:** First look results. ^a^ values for only those participants with 2 or more trials starting on the face; ^b^ proportion calculated out of all trials where gaze was on face at the beginning of the actress’ gaze shift; ^c^ HR-TYP > HR-ASD, HR-TYP > HR-ATYP.

**S4. A closer replication of Bedford et al., 2012: Referent dwell time, congruent first looks only**

This analysis matched exclusion criteria as closely as possible to those used in Bedford et al. (2012) to assess whether the data in this cohort (BASIS Phase 2) replicated outcome group referent dwell time differences found in the previous cohort (BASIS Phase 1). In that study, dwell times were calculated only for trials in which (1) infants looked at the face before the gaze shift and (2) infants made a congruent first look to the gazed-at object (referent). We therefore included only those trials in which infants were looking at the face within 200ms (2750-2950ms) of the start of the gaze shift. However, in the Bedford et al. study, the actress performed only one gaze shift per trial with gaze maintained on the referent object until the end of the trial, whereas in the current study the actress performed 3 short gaze shifts. We therefore completed analyses on two time periods: firstly, during the first gaze shift only, which excluded multiple gaze shifts but meant the total time considered was significantly shorter than that in Bedford et al. (2650ms duration, compared to 6000ms in Bedford et al.); secondly, during all gaze shifts, from the start of the first gaze shift until the end of the trial (8917ms duration). Participants who provided one or more trials entered the analyses (99 participants: 20 LR, 49 HR-TYP, 21 HR-ATYP, 9 HR-ASD). There were no differences between outcome groups in the number of trials contributing (p =.238), age (p = .444), data quality during the first gaze shift (p = 0.195) or in data quality during all gaze shifts (p = .128).

We analyzed the referent dwell time scaled by overall screen dwell time (R/(R+D+O+F)) using the Generalized Estimating Equation (GEE) approach used by Bedford et al. (2012). The analysis used a Gaussian model with identity link (participant id) between predictors and expected proportions with an unstructured working correlation matrix. AOI was a within-participant and outcome group a between-participant variable.

For the first gaze shift only, there were no significant differences between outcome groups [Waldχ^2^ (3) = 1.186, p = .756], significant effects of age [Waldχ^2^ (1) = .352, p = .553] or data quality [Waldχ^2^ = .290, p = .590]; see **Figure S2**. For dwell time across all gaze shifts, there were no significant effects of age [Waldχ^2^ (1) = .078, p = .780] or data quality [Waldχ^2^ = .022, p = .881]. However, there were significant differences between outcome groups [Waldχ^2^ (3) = 13.669, p = .003]. Bonferroni corrected pairwise comparisons indicated HR-ATYP infants looked at the referent significantly less than HR-TYP (p = .002).


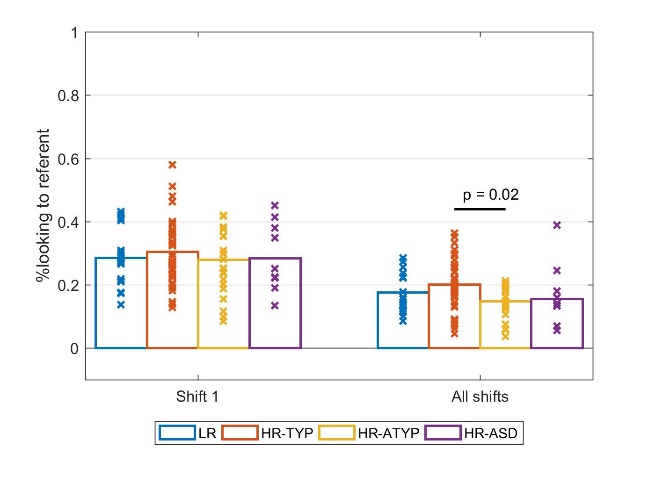


**Figure S2:** Referent dwell time (scaled by screen time) for first gaze shift only and all shifts (whole trial from start of first gaze shift)

*S4.1* *Discussion*

Unlike in the main manuscript (section “Attention to the Referent Relative to Screen Time”), there were no significant differences in referent dwell time between infants with later ASD (HR-ASD) and other outcome groups. The loss of 3 HR-ASD participants, resulting from applying stricter exclusion criteria, may have under-powered the analyses (only 9 HR-ASD infants contributed data compared to 12 in the main manuscript). Further, it is worth noting that for the whole trial comparison, the difference between the larger HR-ATYP and HR-TYP groups did reach significance as it did in Bedford et al. (2012).

**S5. Associations between attention distribution during teaching trials and phenotypic measures for HR only cohort**

|  | | **referent vs. distractor** | **referent** | **distractor** | **face** |
| --- | --- | --- | --- | --- | --- |
| **ADI 36 mo.** | **Social** | .010 | -.054 | -.018 | .023 |
|  | **Comm.** | -.007 | -.152 | -.069 | .101 |
|  | **RRB** | .030 | -.082 | -.066 | .048 |
| **ADOS 36 mo.** | **Social affect** | -.016 | -.113 | -.070 | .124 |
|  | **RRB** | .028 | -.076 | -.075 | .074 |
| **SRS 36 mo.** | **T-score** | .008 | -.022 | .037 | -.021 |
| **CDI words understood** | **15 mo.** | -.031 | .179* | .137 | -.146 |
|  | **24 mo.** | -.012 | .138 | .059 | -.105 |
| **MSEL Verbal** | **15 mo.** | .094 | .218 | .106 | -.159 |
|  | **36 mo.** | -.063 | .265* | .166 | -.282* |
| **MSEL  Non-Verbal** | **15 mo.** | -.075 | .126 | .130 | -.103 |
|  | **36 mo.** | -.094 | .191 | .137 | -.258* |
| **MSEL Total** | **15 mo.** | .030 | .213 | .111 | -.161 |
|  | **36 mo.** | -.087 | .241 | .157 | **-.287** |
| **MSEL Total** | **36 mo. ^$^** | -.119 | .167 | .106 | -.252* |

**Table S2:** HR siblings only cohort, associations between gaze following and attention distribution dwell time measures during teaching trials and phenotypic measures. ADI (The Autism Diagnostic Interview – Revised); ADOS (The Autism Diagnostic Observation Schedule), values used were calibrated severity scores; SRS (Social Responsiveness Scale), value was the total t-Score; CDI words understood (MacArthur-Bates Communicative Development Inventory) was derived by summing words understood only and words understood and spoken; MSEL (Mullen Scales of Early Learning), MSEL Verbal combined verbal subscales (receptive and expressive language), MSEL Non-Verbal combined two non-verbal subscales (visual reception and fine motor), MSEL ELC (Early Learning Composite) combined those four subscales. Where both values were normally distributed, parametric Pearson’s r is reported (shaded) otherwise where one or both variables were not normally distributed, values are Kendall’s tau (unshaded). All values are uncorrected for multiple comparisons. * at p <= 0.01 level; bold values indicate where correlations remained significant after correlation for multiple comparison (p <=. 004). ^$^ accounting for 15 mo. MSEL.

**S6. Associations between first look and phenotypic measures**

*S6.1 Whole cohort*

There were no associations between first look measures and continuous measures of ASD symptoms. However, the first look referent distractor comparison negatively associated with concurrent CDI, with greater first looks to the referent relative to the distractor associated with poorer language. Also, first looks to the distractor, scaled by all trials starting on the face, positively associated with concurrent CDI whereas first looks to the referent did not. Thus, first looks to distractors appear to be the more predictive measure. These associations support the argument made previously linking more first looks to distractors with better memory (see section S3). In the HR siblings only sample, the associations also extended to the 24-month CDI suggesting the memory effects present at 15 months may still be present at 24 months for HR siblings.

|  | | **First look**  **referent vs. distractor** | **First look**  **referent/all** | **First look distractor/all** |
| --- | --- | --- | --- | --- |
| **ADI 36 mo.** | **Social** | .174 | .002 | -.154 |
|  | **Comm.** | .140 | -.132 | -.148 |
|  | **RRB** | .138 | .013 | -.117 |
| **ADOS 36 mo.** | **Social affect** | .081 | .031 | .-.079 |
|  | **RRB** | .191 | .081 | -.174 |
| **SRS 36 mo.** | **t-score** | .089 | .044 | -.064 |
| **CDI words understood** | **15 mo.** | **-.226** | .079 | **.225** |
|  | **24 mo.** | -.182 | .085 | .189 |
| **MSEL Verbal** | **15 mo.** | -.132 | .149 | .148 |
|  | **36 mo.** | -.183 | .073 | .209* |
| **MSEL  Non-Verbal** | **15 mo.** | -.039 | .158 | .070 |
|  | **36 mo.** | -.126 | .106 | .129 |
| **MSEL Total** | **15 mo.** | -.097 | .181* | .125 |
|  | **36 mo.** | -.157 | .099 | .178 |

**Table S3:** Whole cohort association between first look during teaching trials and phenotypic measures. ADI (The Autism Diagnostic Interview – Revised); ADOS (The Autism Diagnostic Observation Schedule), values used were calibrated severity scores; SRS (Social Responsiveness Scale), value was the total t-Score; CDI words understood (MacArthur-Bates Communicative Development Inventory) was derived by summing words understood only and words understood and spoken; MSEL (Mullen Scales of Early Learning), MSEL Verbal combined verbal subscales (receptive and expressive language), MSEL Non-Verbal combined two non-verbal subscales (visual reception and fine motor), MSEL ELC (Early Learning Composite) combined those four subscales. One or both variables were not normally distributed hence values are Kendall’s tau. All values are uncorrected for multiple comparisons. * at p <= .01 level; bold values indicate where correlations remained significant after correlation for multiple comparison (p <=. 004).

*S6.2 HR siblings only cohort*

|  | | **First look referent vs. distractor** | **First look**  **referent/all** | **First look**  **distractor/all** |
| --- | --- | --- | --- | --- |
| **ADI 36 mo.** | **Social** | .206 | .032 | -.171 |
|  | **Comm.** | .173 | -.112 | -.171 |
|  | **RRB** | .155 | -.004 | -.130 |
| **ADOS 36 mo.** | **Social affect** | .095 | .044 | -.095 |
|  | **RRB** | .242 | .108 | -.206 |
| **SRS 36 mo.** | **t-score** | .113 | .060 | -.074 |
| **CDI words understood** | **15 mo.** | **-.295** | .037 | **.279** |
|  | **24 mo.** | **-.284** | .032 | **.268** |
| **MSEL Verbal** | **15 mo.** | -.202 | .133 | .213* |
|  | **36 mo.** | -.218* | .062 | .230* |
| **MSEL  Non-Verbal** | **15 mo.** | -.107 | .167 | .140 |
|  | **36 mo.** | -.149 | .116 | .151 |
| **MSEL Total** | **15 mo.** | -.179 | .171 | .203* |
|  | **36 mo.** | -.192 | .090 | .203* |

**Table S4:** HR siblings only cohort, associations between first look during teaching trials and phenotypic measures. ADI (The Autism Diagnostic Interview – Revised); ADOS (The Autism Diagnostic Observation Schedule), values used were calibrated severity scores; SRS (Social Responsiveness Scale), value was the total t-Score; CDI words understood (MacArthur-Bates Communicative Development Inventory) was derived by summing words understood only and words understood and spoken; MSEL (Mullen Scales of Early Learning), MSEL Verbal combined verbal subscales (receptive and expressive language), MSEL Non-Verbal combined two non-verbal subscales (visual reception and fine motor), MSEL ELC (Early Learning Composite) combined those four subscales. One or both variables were not normally distributed hence values are Kendall’s tau. All values are uncorrected for multiple comparisons. * at p <= .01 level; bold values indicate where correlations remained significant after correlation for multiple comparison (p <=. 004).

**References**

Bedford, R., Elsabbagh, M., Gliga, T., Pickles, A., Senju, A., Charman, T., & Johnson, M. H. (2012). Precursors to social and communication difficulties in infants at-risk for autism: gaze following and attentional engagement. *Journal of autism and developmental disorders*, *42*(10), 2208-2218.
Briganti, A. M., & Cohen, L. B. (2011). Examining the role of social cues in early word learning. *Infant Behavior and Development*, 34(1), 211-214.
Fernald, A., Marchman, V. A., & Weisleder, A. (2013). SES differences in language processing skill and vocabulary are evident at 18 months. *Developmental science*, 16(2), 234-248.
Gliga, T., Elsabbagh, M., Hudry, K., Charman, T., Johnson, M. H., & BASIS Team. (2012). Gaze following, gaze reading, and word learning in children at risk for autism. *Child development,* 83(3), 926-938.
Goodman, R., Ford, T., Richards, H., Gatward, R., & Meltzer, H. (2000). The Development and Well-Being Assessment: description and initial validation of an integrated assessment of child and adolescent psychopathology. *The Journal of Child Psychology and Psychiatry and Allied Disciplines*, 41(5), 645-655.
Parish‐Morris, J., Hennon, E. A., Hirsh‐Pasek, K., Golinkoff, R. M., & Tager‐Flusberg, H. (2007). Children with autism illuminate the role of social intention in word learning. *Child development*, 78(4), 1265-1287.
Paulus, M., & Fikkert, P. (2014). Conflicting social cues: Fourteen-and 24-month-old infants' reliance on gaze and pointing cues in word learning. *Journal of Cognition and Development*, 15(1), 43-59.
Pomiechowska, B., & Gliga, T. (2019). Lexical acquisition through category matching: 12-month-old infants associate words to visual categories. Psychological science, 30(2), 288-299.
Rutter, M., Bailey, A., & Lord, C. (2003). The social communication questionnaire: Manual. Western Psychological Services.
Samuelson, L. K., Smith, L. B., Perry, L. K., & Spencer, J. P. (2011). Grounding word learning in space. PloS one, 6(12), e28095.
Schafer, G., & Plunkett, K. (1998). Rapid word learning by fifteen‐month‐olds under tightly controlled conditions. *Child development*, *69*(2), 309-320.
Wass, S. V., Clackson, K., Georgieva, S. D., Brightman, L., Nutbrown, R., & Leong, V. (2018). Infants' visual sustained attention is higher during joint play than solo play: is this due to increased endogenous attention control or exogenous stimulus capture? *Developmental science*, e12667.
Woodward, A. L., Markman, E. M., & Fitzsimmons, C. M. (1994). Rapid word learning in 13-and 18-month-olds. *Developmental psychology*, *30*(4), 553.
Yin, J., & Csibra, G. (2015). Concept-based word learning in human infants. *Psychological science*, 26(8), 1316-1324.
Yu, C., & Smith, L. B. (2013). Joint attention without gaze following: Human infants and their parents coordinate visual attention to objects through eye-hand coordination. *PloS one*, 8(11), e79659.
Yu, C., & Smith, L. B. (2016). The social origins of sustained attention in one-year-old human infants. *Current Biology*, 26(9), 1235-1240.
Yurovsky, D., & Frank, M. C. (2017). Beyond naïve cue combination: Salience and social cues in early word learning. *Developmental science*, 20(2), e12349.
